# Effect of Tyrosol and Farnesol on Virulence and Antibiotic Resistance of Clinical Isolates of* Pseudomonas aeruginosa*

**DOI:** 10.1155/2015/456463

**Published:** 2015-12-29

**Authors:** Shaymaa Hassan Abdel-Rhman, Areej Mostafa El-Mahdy, Mohammed El-Mowafy

**Affiliations:** ^1^Department of Microbiology and Immunology, Faculty of Pharmacy, Mansoura University, Mansoura 35516, Egypt; ^2^Department of Pharmaceutical Sciences, Faculty of Pharmacy, Princess Norah Bint Abdulrahman University, Riyadh 11671, Saudi Arabia

## Abstract

Mixed-species biofilms could create a protected environment that allows for survival to external antimicrobials and allows different bacterial-fungal interactions.* Pseudomonas aeruginosa*-*Candida albicans* coexistence is an example for such mixed-species community. Numerous reports demonstrated how* P. aeruginosa* or its metabolites could influence the growth, morphogenesis, and virulence of* C. albicans*. In this study, we investigated how the* C. albicans* quorum sensing compounds, tyrosol and farnesol, might affect Egyptian clinical isolates of* P. aeruginosa* regarding growth, antibiotic sensitivity, and virulence. We could demonstrate that tyrosol possesses an antibacterial activity against* P. aeruginosa* (10 *µ*M inhibited more than 50% of growth after 16 h cultivation). Moreover, we could show for the first time that tyrosol strongly inhibits the production of the virulence factors hemolysin and protease in* P. aeruginosa*, whereas farnesol inhibits, to lower extent, hemolysin production in this bacterial pathogen. Cumulatively, tyrosol is expected to strongly affect* P. aeruginosa* in mixed microbial biofilm.

## 1. Introduction

Quorum sensing (QS) compounds are signaling molecules that are released by the cells in a manner depending on their population density. In bacteria, the concentration of these compounds is elevated as the number of bacteria increases. Such autostimulatory compounds induce the population to cooperate in diverse behaviors such as bioluminescence, antibiotic production, virulence, biofilm formation, and sporulation [1]. In Gram-negative bacteria, these compounds are usually acyl homoserine lactones, whereas in Gram-positive bacteria, they are often modified peptides [2]. In* Candida albicans*, farnesol (sesquiterpene derivative) [3] and tyrosol (tyrosine derivative) [4] were identified as the main QS compounds in this pathogenic yeast.

Farnesol is a metabolic product of mevalonate/sterol synthetic pathway in eukaryotes [3]. Weber et al., 2008, have shown that farnesol produced* in situ* by planktonic* C. albicans* cultures inhibits biofilm formation [5]. The accumulation of farnesol was found to block the morphological shift from yeast to hyphae form at high cell densities [3], suggesting that* Candida* pathogenesis may be reduced by farnesol [6]. Contrary to farnesol, tyrosol was found to accelerate the morphological transition from yeasts to hyphae [7].

Quorum sensing signals were found not only to modulate single species bacterial and fungal communications, but also to mediate crosstalk between bacteria and the fungus* C. albicans* [8]. Mixed bacterial-*C. albicans* coinfections can be associated with an increase in the severity of the disease. For example, coinoculation of* C. albicans* and* Staphylococcus aureus* in mice was associated with high mortality in comparison with sole inoculation of* S. aureus* [9, 10]. The bacterial-*C. albicans* interactions were not restricted to enhancement of virulence of bacterial species but they were also extended to affect the bacterial resistance to antimicrobial agents. For instance, it was demonstrated that the human pathogen* S. aureus* forms larger biofilms with increased resistance to vancomycin when it is cocultured with* C. albicans* [11]. Biofilms of oral streptococci and* C. albicans* are similarly more resistant to antibiotics than their single species counterparts [8, 10, 12].

A variety of populations of bacteria and fungi colonizes humans. Interactions between these microbial populations can be useful or detrimental to the host either in healthy state or in the settings of disease [8, 10]. A significant proportion of human microbial infections are biofilm associated, wherein the formation of mixed-species biofilms could create a protected environment that allows for survival to external assaults and facilitates different bacterial-fungal interactions [8].

The coexistence of* Pseudomonas aeruginosa* and* C. albicans* is an example of a pathogenic community.* P. aeruginosa* is extremely difficult to treat due to its high intrinsic and adaptive antibiotic resistance, ability to form biofilms in chronic infections, and broad arsenal of virulence factors [13]. Several reports indicate that* P. aeruginosa* and* C. albicans* can coexist in different opportunistic infections [14–18], and a variety of different molecular interactions between these two organisms have been investigated [18–22]. In the last years, both pathogenic organisms have been shown to be involved in device-associated nosocomial infections for almost all types of indwelling devices [23].

Therefore in this study we were interested in investigating the possible effect of* C. albicans* QS compounds, tyrosol and farnesol, on the antibiotic resistance and some of the virulence factors of Egyptian isolates of* P. aeruginosa*.

## 2. Methods

### 2.1. Bacterial Strains and Reagents

The strains of* P. aeruginosa* used in this study were clinical isolates collected from Mansoura University Hospital and obtained from different sources: burn, wound, urine, and pus. All the clinical isolates were collected after approval from the administrative authorities (Research Ethics Committee) in the Faculty of Pharmacy, Mansoura University, Egypt, on 13/10/2014, with the code number 214-70. The isolates were stored at −70°C till use. Farnesol (Sigma number 277541) and tyrosol (Sigma number 79058) were stored at −20°C before preparation of 500 mM stock solution in DMSO or water for farnesol and tyrosol, respectively. Farnesol stock solution was prepared immediately prior to each experiment and it was added to the final concentration just at the time of inoculation, where control cultures received an equivalent amount of DMSO [24]. Muller-Hinton (MH) broth, MH agar, and antibiotic sensitivity disks were obtained from Oxoid.

### 2.2. *P. aeruginosa* Growth Assay upon Farnesol or Tyrosol Treatment

The growth of representative eight Egyptian isolates of* P. aeruginosa* was tested in the presence of different concentrations of tyrosol and farnesol according to [25] with little modification. Briefly, 2 × 10^5^ cells/mL of* P. aeruginosa* were inoculated in MH broth supplemented with different concentrations of farnesol (10, 50, 100, and 200 *μ*M) or tyrosol (1.2, 2.5, 5, 10, 20, 40, and 80 *μ*M), employing 96-well plates in a final volume of 200 *μ*L. In case of farnesol containing wells, the final volume of DMSO was 1% the total volume in each well. Farnesol and tyrosol free controls were supplemented with 1% DMSO and water, respectively. Cultures were allowed to grow at 37°C for 16 hours. The growth of the cells was determined by measuring the optical density (OD) at 600 nm using the absorbance microplate reader, BioTek ELx800. All experiments were carried out in triplicate.

The effect of farnesol and tyrosol on cell viability was also estimated by colony counting. The cell densities, incubation conditions, and the concentrations of tyrosol and farnesol were followed as previously mentioned in the measurement of OD_600 nm_ in the 96-well plates except that the culture volume was adjusted to 2 mL shaking at 150 rpm. After incubation, the cells were harvested by centrifugation at 4000 rpm for 5 min and washed with MH broth before being plated on MH agar for at least 24 h at 37°C to determine their viability. The survival rate of these cultures, expressed as colony forming units (CFU), was compared to farnesol and tyrosol free cultures.

### 2.3. Disk Diffusion Assay

The susceptibility of* P. aeruginosa* strains to a number of clinically important antibiotics was investigated in the presence of farnesol or tyrosol. Cultures were grown for overnight at 37°C with shaking in MH broth. Aliquots (100 *μ*L) were streaked onto MH agar plates with farnesol final concentration (10 *μ*M) or tyrosol final concentration of (1.2 *μ*M) followed by application of the following antibiotic-impregnated disks (Oxoid Ltd.). Nucleic acid inhibitors (ciprofloxacin [5 *μ*g] and norfloxacin [10 *μ*g]), cell wall synthesis inhibitors (ceftriaxone [30 *μ*g] and cefepime [30 *μ*g]), intermediary metabolism inhibitors (trimethoprim and sulfamethoxazole [25 *μ*g]), and protein synthesis inhibitors (erythromycin [15 *μ*g] and gentamicin [10 *μ*g]) were applied to pseudomonas-seeded agar plates at specified concentration of farnesol or tyrosol. Plates were incubated at 37°C for 18 h, and the zones of inhibition surrounding the antibiotic disks were measured and recorded [26].

### 2.4. Effect of Farnesol and Tyrosol on* P. aeruginosa* Total Proteases and Hemolysin

The effect of farnesol and tyrosol on the production of total protease and hemolysin enzymes of* P. aeruginosa* isolates was performed using previously prepared culture supernatant.

Total protease activity was measured using a casein substrate as previously described [27].

The hemolysin test was carried out by incubating 0.6 mL of fresh sheep red blood cells (2%) with 0.6 mL of bacterial supernatant at 37°C for 2 h. Hemoglobin release was measured at OD_540 nm_. The test was also performed in absence of the bacterial supernatant (negative control) or in presence of SDS (0.2%) instead of the bacterial supernatant (positive control) [28].

### 2.5. Statistical Analysis

Statistical analysis was performed using Student's *t*-test for paired data. Significance was set at 0.1% using Microsoft Excel.

## 3. Results

### 3.1. Farnesol and Tyrosol Inhibit the Growth and Viability of* P. aeruginosa*

**Figure 1:**
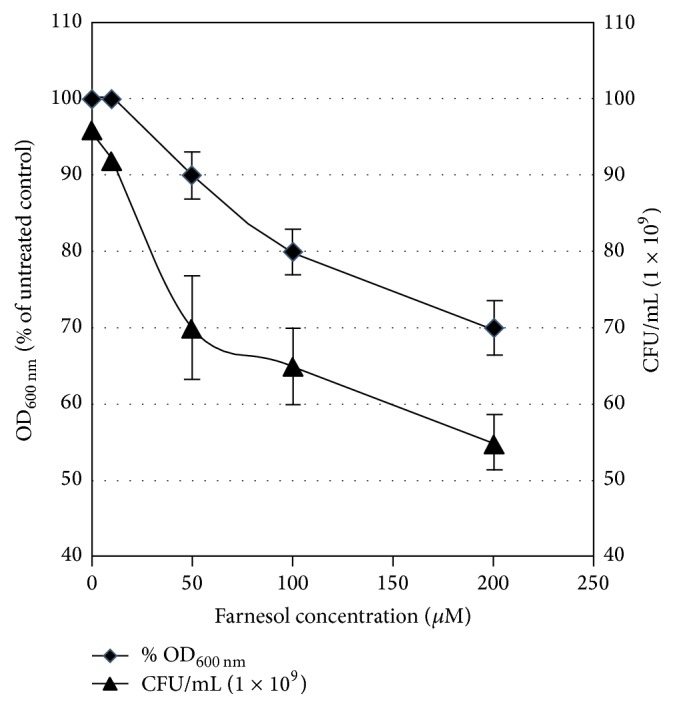
Growth-inhibitory effects of farnesol on cells of* P. aeruginosa* (isolate 1). Cells were grown in MH broth medium containing farnesol at 0, 10, 50, 100, and 200 *μ*M. Cell growth was expressed either as % growth after relating to the untreated cultures (at OD_600 nm_) or as number of cells (CFU) counted after 24 h cultivation on MH agar. Error bars represent the standard deviation from three independent experiments.

**Figure 2:**
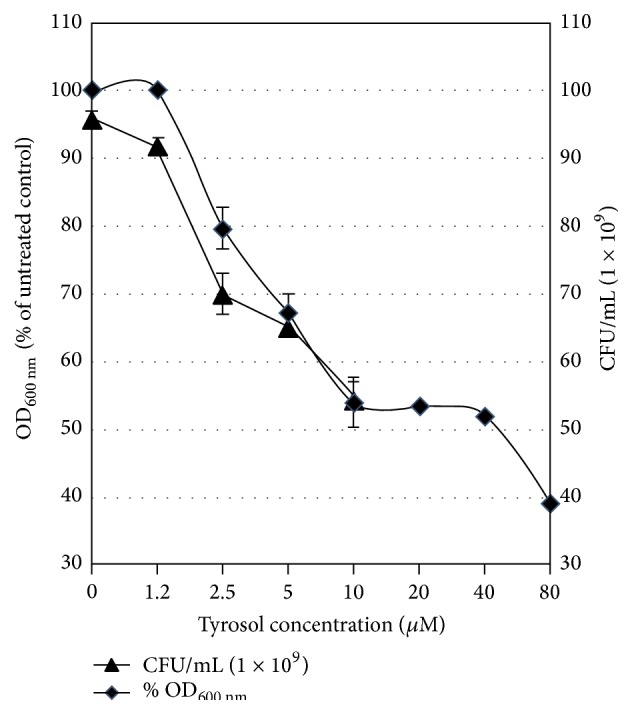
Growth-inhibitory effects of tyrosol on cells of* P. aeruginosa* (isolate 1). Cells were grown in MH broth medium containing tyrosol at 0, 1.25, 2.5, 5, 10, 20, 40, and 80 *μ*M. Cell growth was expressed either as % growth after relating to the untreated cultures (at OD_600 nm_) or as number of cells (CFU) counted after 24 h cultivation on MH agar. Error bars represent the standard deviation from three independent experiments.

In this work we evaluated the influence of the quorum sensing compounds of* C. albicans* (farnesol and tyrosol) on the growth of representative eight Egyptian isolates of* P. aeruginosa*. These results were identical in the eight tested isolates of* P. aeruginosa*. A representative data for the effect of farnesol and tyrosol on one of these isolates (isolate 1) is shown in Figures 1 and 2, respectively. The growth was inhibited after 16 h by 30% or more than 50% in the presence of 200 *μ*M farnesol or 10 *μ*M tyrosol, respectively. On the other side, 1.2 *μ*M tyrosol and 50 *μ*M farnesol either had no effect on growth or inhibited it by 10%, respectively.

Both the percent of growth (expressed as OD_600 nm_) and the number of viable bacterial cells decreased proportionally with farnesol and tyrosol concentrations, suggesting that both compounds have an antibacterial activity against* P. aeruginosa*.

### 3.2. Influence of Farnesol and Tyrosol on the Antibiotic Sensitivity of* P. aeruginosa*

Disk diffusion assay showed no detectable difference in the inhibition zones obtained by the discs of tested antibiotics in presence or absence of either of the two investigated QS compounds. These results were further confirmed by data obtained from broth microdilution experiments (data not shown). These experiments were performed using twofold serial dilutions of three of the most widely administrated broad spectrum antibiotics: ceftriaxone, ciprofloxacin, and gentamicin at concentrations of 64000-0.12, 4000-0.004, and 2000-0.002 *μ*g/mL, respectively. In both disk diffusion and broth microdilution experiments, the effect of tyrosol and farnesol was tested at concentrations 1.2 and 10 that affect the growth yield by not more than 10%.

### 3.3. Effect of Farnesol and Tyrosol on Total Proteases and Hemolysin Production by* P. aeruginosa*

We next examined the effect of farnesol and tyrosol on the production of total proteases and hemolysin. Treating* P. aeruginosa* isolates with farnesol (10 *μ*M) or tyrosol (1.2 *μ*M) caused reduction in the production level of hemolysin (Table 1) and total proteases (Table 2).

Tyrosol significantly inhibited the production of hemolysin (*p* < 0.001) and total proteases (*p* < 0.001) in the investigated isolates. Additionally, farnesol significantly inhibited the production hemolysin (*p* < 0.001) without signification inhibition of total proteases.

## 4. Discussion

The interaction between* C. albicans* and* P. aeruginosa* in mixed communities was reported in many studies. From the side of* P. aeruginosa*, the effect of either the bacterial pathogen itself or its metabolites on* C. albicans* was extensively investigated in numerous studies [11, 18, 29, 30]. Few reports have investigated the effect of coculturing of* C. albicans* or incubation with farnesol on* P. aeruginosa* regarding growth, virulence, and antibiotic resistance of this bacterial pathogen. For example,* C. albicans* has been shown to enhance the production of* P. aeruginosa* phenazine toxins in cocultured colony biofilms [18, 31]. Moreover, the quorum sensing-regulated phenazines in* P. aeruginosa* were stimulated when cultured with* C. albicans* [31]. Farnesol was shown to inhibit the swarming motility in* P. aeruginosa* [32].

In this work we were interested to determine how the* C. albicans* QS compounds (tyrosol and farnesol) might affect the antibiotic sensitivity and production of the virulence factors, hemolysin and total proteases in Egyptian isolates of* P. aeruginosa*. To our knowledge, the effect of tyrosol on* P. aeruginosa* is not yet reported. Care was taken to perform firstly growth studies of a concentration-dependent manner for farnesol and tyrosol. Our results showed that the growth of* P. aeruginosa* was inhibited by more than 50% in the presence of 10 *μ*M tyrosol, while 1.2 *μ*M was the highest concentration that did not exhibit any effect on the growth of* P. aeruginosa*. On the other side, farnesol inhibited the growth of* P. aeruginosa* by 10 and 30% at concentrations of 50 and 200 *μ*M, respectively. Previous report showed that farnesol did not affect the growth of* P. aeruginosa* strain PA14 at a concentration of 250 *μ*M [24]. It is important to mention that the corresponding physiological relevant concentration of tyrosol and farnesol produced by stationary-phase* C. albicans* cultures is about 350–400 *μ*M [4] and 50 *μ*M [5, 33], respectively. This indicates that the* C. albicans* QS molecule, tyrosol, would certainly affect the growth of* P. aeruginosa* in mixed biofilms. Our results of the inhibitory effects of farnesol are consistent with the observed antibacterial effect of farnesol against other bacterial species such as* Staphylococcus aureus* [26],* Streptococcus mutans* [34], and* Staphylococcus epidermidis* [35].

For further investigation of the effect of tyrosol and farnesol on antibiotic sensitivity and the production of hemolysin and total proteases in the isolates of* P. aeruginosa*, subinhibitory concentrations that were not exceeding the corresponding physiological relevant concentration were further used in this study (1.2 and 10 *μ*M for tyrosol and farnesol, resp.).

Jabra-Rizk et al., 2006, have shown that the antibiotic sensitivity in* S. aureus* was increased in the presence of farnesol [26]; they related this synergistic effect to the possible membrane permeabilizing action of farnesol that subsequently increases the sensitivity of* S. aureus* to antibiotics. Indeed, many compounds that are able to increase the sensitivity to antibiotics might have outer membrane permeabilizing action [36]. Our results showed that neither tyrosol nor farnesol, at their subinhibitory concentrations, was found to have any effect on the antibacterial activity of different antibiotics on* P. aeruginosa*. We might also conclude that farnesol and tyrosol do not have membrane permeabilizing action on* P. aeruginosa*, which is in contrast to the membrane permeabilizing action of farnesol for gentamicin in some strains of* S. aureus* reported by Jabra-Rizk et al. [26].

Hemolysin and protease are considered to be important virulence factors of* P. aeruginosa* [37–39] which contribute to the pathogenesis of* Pseudomonas* infection. Our results indicate without doubt that tyrosol and farnesol strongly inhibit the production of hemolysin in almost all of the Egyptian isolates under investigation. Out of the 20 Egyptian isolates of* P. aeruginosa*, 16 strains showed more than 30% inhibition of hemolysin production after incubation with tyrosol (1.2 *μ*M), while in case of farnesol, 14 strains showed the same effect. In comparison with hemolysin results, we observed that tyrosol inhibited to much lower extent the production of total proteases in the same investigated isolates, where 13 stains showed more than 10% inhibition in the production of such virulence factor, whereas no significant inhibitory effect could be observed after incubation of the isolates with farnesol (10 *μ*M). Previous reports demonstrated that farnesol inhibited the virulence factor lipase in* S. aureus* [40]. However, no studies have investigated the effect of tyrosol on any of the bacterial virulence factors.

In conclusion, despite their difference in chemical structure, our results indicate that the* C. albicans* QS compounds, tyrosol and farnesol, inhibit the production of hemolysin by* P. aeruginosa*. Additionally, the production of protease by* P. aeruginosa* was repressed in the presence of tyrosol; however no significant decrease was observed in case of farnesol. Together with our novel observation of the antibacterial activity of tyrosol against* P. aeruginosa*, a suggestion for effective antimicrobial strategy may be broadly applicable to other bacterial species. Therefore, our research highlights the importance of studying effect of* C. albicans* QS compounds on* P. aeruginosa* as an example for bacterial pathogen that can coexist in mixed-species of microbial communities and might play an important role* in vivo*. Further studies are now in progress to identify the mechanism of inhibition of these virulence factors by tyrosol particularly the expression of hemolysin gene.

## Figures and Tables

**Table 1:** Effect of farnesol and tyrosol on hemolysin production in Egyptian isolates of *P. aeruginosa*.

Isolate number	Control	Tyrosol effect		Farnesol effect	
	Hemolysis percent caused by the isolate	Before normalization	After normalization	Before normalization	After normalization
1	23.5	4.8175	−79.5	6.4625	−72.5
2	12.3	1.1931	−90.3	2.46	−80
3	27.4	22.8242	−16.7	24.4956	−10.6
4	60.7	11.6544	−80.8	33.8099	−44.3
5	59.8	3.887	−93.5	17.94	−70
6	34.1	22.7447	−33.3	30.008	−12
7	72.3	19.521	−73	7.5915	−89.5
8	38.8	30.458	−21.5	29.4104	−24.2
9	89.9	36.4095	−59.5	21.576	76
10	100	1	−99	25	−75
11	10	9	−10	9	−10
12	79.4	6.352	−92	29.2192	−63.2
13	71	20.519	−71.1	47.357	−33.3
14	22.3	15.2978	−31.4	12.9117	−42.1
15	50.5	13.5845	−73.1	14.7965	−70.7
16	81.1	7.3801	−90.9	20.275	−75
17	7.6	6.802	−10.5	7.448	−2
18	92.3	27.69	−70	50.765	−45
19	90	21.51	−76.1	34.65	−61.5
20	31.7	21.4609	−32.3	10.1123	−68.1

The values in the control column represent the levels of hemolysin production by the isolates (SD = 30.6), indicated by hemolysis percent, without addition of farnesol and tyrosol. The values in the columns of tyrosol and farnesol stand for either nonnormalized values (SD = 10.2 and 13.5 for tyrosol and farnesol, resp.) or normalized values. The latter values indicate the percent of either decrease (negative values) or increase (positive values) in the production of hemolysin after normalization to untreated samples (control). All values represent the mean of three independent experiments.

**Table 2:** Effect of farnesol and tyrosol on total protease production in Egyptian isolates of *P. aeruginosa*.

Isolate number	Control	Tyrosol effect		Farnesol effect	
	Total protease production by the isolate (U/mL)	Before normalization	After normalization	Before normalization	After normalization
1	107	75.6	−29.3	89	−16.8
2	95	61	−35.8	57.5	39.5
3	100	88.5	−11.5	80	−20
4	60.8	57.3	−5.6	10.6	82.5
5	198.8	154.6	−22.2	102	−48.7
6	22	21	−4.5	22	−0.1
7	76	60.8	−20	64	−15.8
8	102.8	85.9	−16.4	102	−0.8
9	128.9	118.2	−8.3	96	−25.5
10	147.9	115.4	−22	121.8	−17.6
11	65.5	54.2	−17.3	65	0.8
12	127	76.4	−39.8	165.4	−30.2
13	33.5	46.4	38.5	32	−4.5
14	94.5	78.4	−17	75.5	20.1
15	160	91.3	−42.9	143	−10.6
16	63.7	61.8	−3	62	−2.7
17	90	70.7	−21.4	101.7	13
18	109	100.8	−7.5	99	−9.2
19	130.7	123.8	−5.3	82	−37.3
20	163	101.5	−37.7	150	−8

The values in the control column represent the levels of total protease production by the isolates (SD = 44.3), as determined in [27], without addition of farnesol and tyrosol. The values in the columns of tyrosol and farnesol stand for either nonnormalized values (SD = 40 and 40.5 for tyrosol and farnesol, resp.) or normalized values. The latter values indicate the percent of either decrease (negative values) or increase (positive values) in the production of total protease after normalization to untreated samples (control). All values represent the mean of three independent experiments.
